# The critical impacts of cytokine storms in respiratory disorders

**DOI:** 10.1016/j.heliyon.2024.e29769

**Published:** 2024-04-17

**Authors:** Shahana Riyaz Tramboo, Ahmed M.E. Elkhalifa, Syed Quibtiya, Sofi Imtiyaz Ali, Naveed Nazir Shah, Syed Taifa, Rabia Rakhshan, Iqra Hussain Shah, Muzafar Ahmad Mir, Masood Malik, Zahid Ramzan, Nusrat Bashir, Shubeena Ahad, Ibraq Khursheed, Elsharif A. Bazie, Elsadig Mohamed Ahmed, Abozer Y. Elderdery, Fawaz O. Alenazy, Awadh Alanazi, Badr Alzahrani, Muharib Alruwaili, Emad Manni, Sanaa E. Hussein, Ezeldine K. Abdalhabib, Showkat Ul Nabi

**Affiliations:** a Preclinical Research Laboratory, Department of Clinical Veterinary Medicine, Ethics & Jurisprudence, Sher-e-Kashmir University of Agricultural Sciences and Technology (SKUAST-Kashmir), Srinagar, J&K, 190006, India; b Department of Public Health, College of Health Sciences, Saudi Electronic University, Riyadh, 11673, Saudi Arabia; c Department of Haematology, Faculty of Medical Laboratory Sciences, University of El Imam El Mahdi, Kosti, 1158, Sudan; d Department of General Surgery, Sher-I-Kashmir Institute of Medical Sciences, Medical College, Srinagar, 190011, Jammu & Kashmir, India; e Department of Chest Medicine, Govt. Medical College, Srinagar, 191202, Jammu & Kashmir, India; f Department of Clinical Biochemistry, University of Kashmir, Srinagar, Jammu & Kashmir, 190006, India; g Department of Zoology, Central University of Kashmir, 191201, Nunar, Ganderbal, Jammu & Kashmir, India; h Pediatric Department, Faculty of Medicine, University of El Imam El Mahdi, Kosti, 1158, Sudan; i Department of Medical Laboratory Sciences, College of Applied Medical Sciences, University of Bisha, Bisha, 61922, Saudi Arabia; j Department of Clinical Chemistry, Faculty of Medical Laboratory Sciences, University of El Imam El Mahdi, Kosti, 1158, Sudan; k Department of Clinical Laboratory Sciences, College of Applied Medical Sciences, Jouf University, Al-Qurayyat, Saudi Arabia

**Keywords:** Cytokine storm, SARS-CoV-2, Respiratory diseases, Biomarkers, and immune response

## Abstract

Cytokine storm (CS) refers to the spontaneous dysregulated and hyper-activated inflammatory reaction occurring in various clinical conditions, ranging from microbial infection to end-stage organ failure. Recently the novel coronavirus involved in COVID-19 (Coronavirus disease-19) caused by SARS-CoV-2 (Severe Acute Respiratory Syndrome Coronavirus 2) has been associated with the pathological phenomenon of CS in critically ill patients. Furthermore, critically ill patients suffering from CS are likely to have a grave prognosis and a higher case fatality rate. Pathologically CS is manifested as hyper-immune activation and is clinically manifested as multiple organ failure. An in-depth understanding of the etiology of CS will enable the discovery of not just disease risk factors of CS but also therapeutic approaches to modulate the immune response and improve outcomes in patients with respiratory diseases having CS in the pathogenic pathway. Owing to the grave consequences of CS in various diseases, this phenomenon has attracted the attention of researchers and clinicians throughout the globe. So in the present manuscript, we have attempted to discuss CS and its ramifications in COVID-19 and other respiratory diseases, as well as prospective treatment approaches and biomarkers of the cytokine storm. Furthermore, we have attempted to provide in-depth insight into CS from both a prophylactic and therapeutic point of view. In addition, we have included recent findings of CS in respiratory diseases reported from different parts of the world, which are based on expert opinion, clinical case-control research, experimental research, and a case-controlled cohort approach.

## Highlights

• Cytokine storm is the outcome of respiratory illnesses such as MERS, SARS, and COVID-19.
• Patients with cytokine storms exhibit mild to severe clinical manifestations.
• Immune pathways such as JAK/STAT, mTOR, ERK, and TRAF/NF-κB are stimulated in respiratory illness.
• Cytokine storm can be early controlled using immunomodulators and cytokine antagonists.

## 1 Introduction

Cytokine storm refers to a dysregulated and excessive immune response characterized by the release of a large number of pro-inflammatory cytokines into the bloodstream. This phenomenon can have severe consequences and is associated with various medical conditions, including viral infections, autoimmune diseases, and certain therapies [1,2]. The COVID-19 epidemic has underlined the significance of a robust host immune system and deleterious consequences observed in immunological dysregulation [1,2]. Throughout the body, a variety of cells release tiny glycoproteins called cytokines that have a variety of functions prominent among them including cell proliferation, cellular differentiation, autocrine, paracrine, and/or endocrine functions, as well as influencing immune and inflammatory responses [3]. The abnormal release of cytokines in response to infection or stimulation, also known as hypercytokinemia, can result in life-threatening immune dysregulation [4]. Such systemic inflammatory disorders are characterized by an elevated number of circulating cytokines, and hyper-activation of immune-cells, all of which cause multiple organ failure if not treated properly and the pathological condition has been referred to as cytokine storm (CS) [5]. The phrase "cytokine storm" was coined by Ref. [6] during a medical literature discussion on graft versus host disease. The perplexing etiology of COVID-19 is marked by significantly enhanced cytokine production which results both in considerable mortality and grave disease progression [6] The increased case fatality of the recent SARS-CoV-2 pandemic is attributed to uncontrolled and dysregulated cytokine production which results from severe Acute Respiratory Distress Syndrome (ARDS) symptoms, which further causes significantly higher mortality rate in these patients [7]. Studies across the globe have reported that activation of immune cells by various viruses that infect the pulmonary system promotes secretion of a diverse set of cytokines which enhances endothelial-vascular permeability and allows blood cells and fluid to migrate into the alveoli henceforth causing dyspnea and respiratory failure [[7], [8], [9], [10], [11], [12]]. In addition to SARS-CoV-2 viruses that induce CS include SARS-CoV-1, H1N1 influenza, H5N1 influenza, Ebola infections, and Influenza Band Parainfluenza virus, [13]. Similarly, non-infectious diseases, such as Graft-versus-host disease (GvHD) can also result in CS [14]. These viruses cause the release of numerous cytokines and chemokines after infecting epithelial cells of the lungs which stimulate alveolar macrophages. Recently, studies have found that the release of viral nucleic acids causes sensitization/activation of immune cells [15]. CS storm has been observed in a wide spectrum of diseases and different authors have reported the alteration in biochemical and clinical presentation of disease (Table 1). Owing to the catastrophic outcomes associated with CS, there is an urgent need to have an in-depth understanding CS, henceforth the study aims to provide insights (i) to provide an understanding about potential risk/etiological factors responsible for initiation of CS in wide spectrum of respiratory diseases. (ii) Critical impact of CS on initiation, progression and outcomes of respiratory disorders (iii) Evaluate the specific cytokines involved CS and their potential to serve as surrogate and endpoint biomarkers for disease severity and prognosis. (iv) potential therapeutic regimens available for targeting CS to mitigate inflammation and improve patient outcomes in respiratory disorders. (v) Enhance understanding of the interplay between cytokine storms and other pathological processes.

**Table 1:** Manifestation of cytokine storm in various infectious diseases with special emphasis on diagnostic criteria for identification of CS.

S.No	Disease	Criteria	References
1)	Viral Haemorrhagic Fever	Haemorrhage, disseminated intravascular coagulation, Immune suppression, higher levels of Ferritin and C-Reactive Protein	[11,12], 205
2)	Dengue Virus	Significantly elevated levels of vascular endothelial growth factor alpha (VEGF-α), TNF-α and IL-6. Immune suppression	[16]
3)	Influenza Virus Infection	Increased production of IL-1α, IL-6, and TNF-α. Increased infiltration of immune cells inside pulmonary parenchyma. Hyper activation of inflammatory pathway, higher levels of Ferritin and C-Reactive Protein	[17,18,19]
4)	Bacteria Induced CS	Increased levels of TNF-α, interluekins chemokines and G-CSF,	[20,21[51,185,190]
5)	SARS-CoV-1	Elevated levels of IL-8, IL-1β, TNF-α, and IL-6 in alveolar lavage and blood plasma and significant Lymphopenia, higher levels of Ferritin and C-Reactive Protein	[8,204]
6)	MERS-CoV-1	Significantly higher levels of procalcitonin, and IL-6 being a prominent indicator of CS. The main differentiating factors includes absence of hyperferritinemia and absence of any significant elevation in C reactive protein levels	[ [20,[18], [22], [23], [24], [25], [26], [27], [28]], 202]
7)	Macrophage Activation Syndrome	Ferritin levels above the threshold level of 10,000 ng/mL. Similarly the markers like neopterin, soluble CD163, and soluble CD25 were found to be elevated.	[29,30,31]
8)	SARS-CoV-2	i. Clinical signs suggestive of COVID-19 and positive for RT-PCR for SARS- CoV-2. ii. GGO in high resolution CT Scan iii. BUN: Creatinine ratio greater than 29 iv. ALT, AST, D-Dimers, LDH and Troponin-I levels above the threshold levels. v. Neutrophil Abs above >11.4 K/mm^3^, vi. ferritin and C Reactive protein levels above threshold levels of >250 ng/mL and >4.6 mg/dL respectively.	[ [13,32,33,[34], [35], [36]], 201]

## 2 Methodology

For drafting the present review, we searched Medline, Pubmed, Elsevier, clinicaltrail.gov.in, and Google Scholar with the words COVID-19, cytokine storm, clinical trials, and a combination of these words. We followed the referred reporting in reviews and meta-analysis, furthermore, for the present study we searched for a database from the World Health Organization clinical trials registry (WHO) for the collection of data we used the technique of snowballing to include only relevant studies in the present study. Search design was followed to include all the published papers on COVID-19-associated CS. A total of 2398 papers were found to deal with COVID-19, out of these 1753 papers were irrelevant for drafting present review articles and they were excluded. From the remaining 645 papers, 456 were found to deal with COVID-19 only and in these papers, there was nothing relevant regarding cytokine storm so they were also excluded, from the remaining 189 papers we drafted in the current review article.

### 2.1 Inclusion/exclusion criteria

#### 2.1.1 Inclusion criteria

i. The present study incorporated case studies/series, meta-analyses/systemic reviews, observational studies (retrospective/prospective), and clinical trials (randomized/non-randomized) that featured confirmed diagnoses of CS and respiratory disorders.
ii. Clinical cases with a confirmatory diagnosis of respiratory disorder and concurrently showing pathognomonic features of CS.
iii. Published Studies/records dealing with the respiratory disorder and concurrent incidence of CS.

#### 2.1.2 Exclusion criteria

i. From the current study, we excluded studies where the confirmatory diagnosis of either the respiratory disorder or CS was not reported, and clinical and biochemical presentation was not documented.
ii. Studies where standard diagnostic protocol/procedures were not adopted for diagnostic criteria established for respiratory disorder and CS.
iii. Unpublished data, thesis, preprints, and studies published in language other than English language were excluded from the drafting of the current manuscript.
iv. Editorial comments, Newspaper writing, personal suggestions, and incomplete data were excluded from the current study.

## 3 Clinical features and clinical abnormalities of CS in pulmonary diseases

**Fig. 1:**
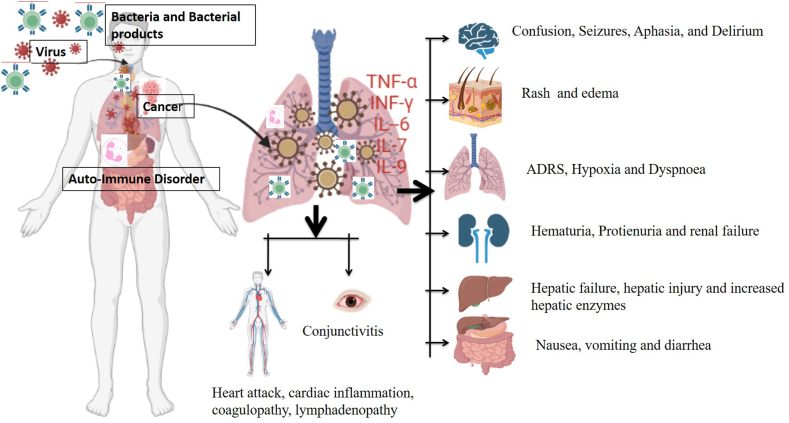
Clinical manifestation of cytokine storm, cytokine storm involves multiple organ failure mediated by hyper-activation of inflammatory pathways. The wide range of clinical abnormalities observed in CS varies from patient to patient based on the severity of the disease.

Cytokines play vital role in progression, and outcome of the pulmonary diseases. After the disease's degenerative processes begin, clinical symptoms become more complicated and complex in manifestation [11]. Because of the simultaneous manifestation of hyper inflammation, hemostasis disruption, and reduced platelet counts, CS may result in a significant risk of spontaneous bleeding [1]. A range of illnesses, including kidney failure, acute hepatitis, and stress-related cardiomyopathy can result in advanced stages of CS [12] (Fig. 1). Similarly, renal failure can cause leaky capillary syndrome which mimics the changes found in cancer patients undergoing therapeutic intervention using high-dose interleukin-2 [13]. The cytokine storm's neurotoxic consequences are usually delayed, taking several days to manifest; these findings are in concurrence with the neurologic side effects of T-cell immunotherapy known as immune effector cell-associated neurotoxicity syndrome [14]. Clinical manifestation in CS differs because of the underlying causes and severity as indicated by nonspecific inflammatory indicators such as C-reactive protein (CRP) [15]. High triglyceride levels, as well as thrombocytopenia, leukopenia, leukocytosis, anemia, elevated acute phase proteins, and other blood-count abnormalities, are considered biochemical mediators which cause dysregulation of immune cum inflammatory pathways which henceforth results in positive feedback for progression of CS [37]. Serum levels of interferons, chemokines, interferon-γ, and other pro-inflammatory markers' as well as T-cell activation factors are all elevated in CS. In CS induced by therapeutic CAR T-cell therapy, serum IL-6 levels were observed to be significantly elevated [38,39]. Furthermore, the levels of IL-6 were observed to be elevated in individuals affected by SARS and other diseases experiencing severe manifestations of the illness [32].

## 4 Pathophysiological characteristics of pulmonary CS

In respiratory illnesses, cytokine storm generates a variety of functional alterations which are manifested as clinical signs of the cytokine storm. Cytokines have systemic effects and can harm important organ systems at certain high levels henceforth rendering them potentially harmful [40]. Excessive production of cytokines might harm vital organs, potentially outweighing the benefits of an immune response [1]. Regulatory cells, as well as decoy receptors for pro-inflammatory cytokines like IL1RA and anti-inflammatory cytokines like IL-10, suppress inflammatory cell populations; henceforth limiting cytokine-induced immune hyper-activation [1]. Hyper-immune activation occurs occasionally as a consequence of dysregulation or increased amplitude of the immune system, to exemplify CAR T-cell therapy, primary hemophagocytic lymph histiocytosis (HLH) [41]. In each of these conditions, feedback mechanisms that would otherwise stop hyper-inflammation are rendered ineffective which results in overproduction of inflammatory cytokines. Owing to the emergence of COVID-19-associated cytokine storm researchers have had an opportunity to distinguish COVID-19 CS and other forms of CS, COVID-19 associated CS involves the release of the wide spectrum of pro-inflammatory cytokines both in quantity and quality compared to other forms of CS [42], henceforth in COVID-19 CS, there is aggressive manifestation of disease spectrum. Lymphopenia is less frequently encountered in other forms of CS compared to COVID-19 CS where lymphopenia constitutes the characteristic finding [43]. Furthermore, compared to other bacterial CS the therapeutic interventions required in COVID-19 CS are more aggressive and need to be more precise in targeting potential targets of the CS. Although the pathogenic pathway involved in COVID-19 CS has not been established based on the analogy drawn from earlier outbreaks like MERS-CoV, it can be postulated that viral entry through ACE-2 receptor and release of the RNA and other components of RNA like double-stranded RNA and PAMPs (pathogen-associated molecule pattern (PAMP) interact with pattern recognition receptors (PRRs) of host cells which results in over expression of pro-inflammatory cytokines, especially IFN-1 [44]. The hyper-inflammatory response impairs viral clearance and causes a cascade of reactions which further results in the production of more pro-inflammatory cytokines by activation of local pulmonary innate response [45]. These pro-inflammatory cytokines attract more inflammatory cells and immune cells, hence causing activation of adaptive immune response with the recruitment of CD4^+^ and CD8^+^ T cells, which results in persistent inflammatory response that activates emergency granulopoiesis, macrophage activation, and erythro-phagocytosis which damages pulmonary tissue architecture. Erythro-phagocytosis results in anemia, capillary leakage, and activation of the intrinsic pathway of coagulation which results in the formation of emboli in the blood vascular system [46]. Furthermore, in CS natural regulatory systems like Tregs which produce IL-10 and TNF-β are down-regulated by excessive production of pro-inflammatory cytokines. Based on these findings’ researchers have proposed that in COVID-19 CS an unfortunate event of uncontrolled immune response culminates in a hyper-inflammatory response [15].

## 5 Criteria for diagnosis and recognition of CS

The criteria for recognition and diagnosis of CS constitute an important topic of discussion among researchers and the healthcare community. Various clinical, biochemical, and radiographic abnormalities are peculiar to CS patients and some of them are pathognomonic for diagnosis of CS [6,11] (Table 2, Table 3).

**Table 2:** Diagnostic criteria proposed by researchers for early identification of CS in COVID-19 patients.

S.no	Diagnostic Criterion of cytokine storm in COVID-19 patients	References
1)	Serum albumin levels less than 2.87 mg/mL, blood lymphocyte count less than 10.2 % and absolute neutrophil count more than 11.4 × 10^3^/mL	[33,47,36]
2)	Significantly higher values of SPO_2_/FiO_2_; Neutrophil/Lymphocyte and cytokines/chemokines are more predictive for COVID-19-CS.	[13,35[13,188, 196]
3)	D-dimer concentrations above 1.5 μg/mL and concurrent incidence of thrombi-embolism is highly predictive of COVID-19 CS. These parameters were found to have high sensitivity and high specificity.	[ [[48], [49], [50], [51]], 198]
4)	Sepsis-induced coagulopathy score of significantly higher with values being more or equal to 4 with concurrently elevated levels of D-Dimer	[51,34]
5)	ALT levels more than 60 IU/L, AST levels more than 87 IU/L, D-Dimer levels more than 4930 ng/mL, LDH levels more than 416 U/L and levels of troponin-I more than 1.09 ng/mL	[33,21]
6)	Ground glass appearance, thickening of alveolar walls, presence of pleural fluid and collapsing of alveoli	[40,41]
7)	Screening for hyper-inflammatory markers and H Score	[1]
8)	D-Dimer levels significantly elevated with fibrinogen levels above 2.0 g/L	[50,52]
9)	Anion gap less than 6.8 mmol/L, significantly higher levels of sodium, potassium and blood urea nitrogen. Levels of ferritin >250 ng/mL and C-Reactive protein >4.6 mg/dL	[33,53]
10)	Significant increase in levels of D-Dimer and prothrombin time greater than 3.0 s and aPTT >5 s	[[50], [51], [54]]
11)	Post-mortem findings of spleen atrophy, visceral haemorrhages and hepatomegaly in COVID-19 patients	[50,55,56]
12)	Severe lymphopenia with pronounced reduction in CD4^+^ and CD8^+^ T cell levels with up regulation of exhaustion markers like NKG2A	[48]

**Table 3:** Role of various pro-inflammatory and inflammatory mediators in COVID-19-associated CS.

Pro-inflammatory cytokine	Role in cytokine storm	Reference
*IL-6*	Causes activation of JAK/STAT signalling pathway which results in hyper-activation of inflammatory cum immune pathways. Causes transformation and maturation of CD8^+^ T and B cells	[[57,58], 202]
*IFN-γ*	Activates JAK/STAT pathway and causes proliferation and sensitization of macrophage, NK, and T cell.	[[59], [60], [61],198]
*TNFα*	TNFα acts as activator of NF-κB which causes up-regulation of the inflammatory and apoptotic genes in immune cells	[[[55], [62], [63], [64], [65], [66]], 197]
*IL-1β*	NLRP3 inflammasome causes cleavage of IL-1βprecursor to active form of IL-1β which provides positive feedback for NF-κB activation which initiates the inflammatory cascade.	[[67], 190–193, 201]
*IL-2*	Significantly lower levels of IL-2 have been observed in other related coronaviruses. Lymphopenia observed in COVID-19-CS have been attributed to lower levels of IL-2	[68,69]
*IL-7*	Significantly higher levels of IL-7 have been attributed to feedback mechanism to Lymphopenia	[ [68],200]
*IL-10*	IL-10 acts as potent immune regulator and is produced by CD8^+^ T cells and Tregs. It regulated immune function by autocrine and paracrine manner by acting on innate immune system.	[[68,70,71,72,69]199]
*IL-12*	Acts as an important immune regulator and acts and causes proliferation of Th1 and Th17 cells and cause activation of diverse set of immune cum inflammatory cells, henceforth contributes in CS.	[7,68,71,34]
*IL-17*	Causes recruitment of immune cells to site of inflammation and promotes expression of pro-inflammatory genes via pleiotropic effect. These cytokines have been reported to cause tissue modelling.	[68,198]
*GM-CSF*	Under normal circumstances *GM-CSF* maintain integrity of alveolar epithelium and provide local anti-bacterial micro-environment. Under hyper inflammation *GM-CSF* causes myelopoiesis and recruits myeloid cells to site of infection/injury, henceforth maintains sustained inflammatory process.	[73,17,195]

## 6 Radiological findings as criteria for CS diagnosis

Although radiological abnormalities observed in patients with pulmonary form of cytokine storm require further standardization and verification in COVID-19 patients concurrently affected with cytokine storm demonstrate gross and microscopic findings which typically represent a hyper-inflammatory state [74]. The most common findings observed in these patients include ground-glass opacities which may be especially in subpleural, peripheral, and bilateral in presentation [75], significantly increased width of pleura [76], ill-defined margin of the pulmonary architecture [77], alveolar consolidation [48] and interlobular thickening [49]. The increase production of IL-1β induces the production of pulmonary exudates which imparts ground glass appearance to lung parenchyma. The cytokine storm operates in chronological order within the pulmonary tissue; up to 3 days of initiation of cytokine storm lung parenchyma appears almost normal, and from the 3–9th day ground glass appearance of pulmonary tissue becomes evident [50,51]. During the later stage of the cytokine storm interlobular thickening occurs and a fibrous strip develops inside the lung parenchyma [54].

## 7 Biochemical alterations as criteria for CS diagnosis

In recent case series, some of the biochemical alterations have been observed in patients with CS [75]. It has been observed that patients with CS have a higher incidence of hypercoagulability which subsequently results in vascular thrombosis mostly observed in younger age groups of patients [78]. Among the various biochemical markers considered D-dimer levels of >1.5 μg/mL had high sensitivity and specificity in the prediction of hypercoagulability [79]. Concurrently, patients with a serum D-dimer level greater than 1 μg/mL were found to have higher mortality rates compared to those with a serum D-dimer level below this threshold [80]. Similarly, Pulmonary thromboembolism (PE) has been reported by Computed Tomography pulmonary angiogram (CTPA) in patients with high D-dimer levels [9,80]. Furthermore, in these patients prothrombin time was reported to be > 3.0 s with prolonged aPTT >5 which indirectly indicates thrombotic complications suggestive of bleeding risk owing to consumption of coagulation factors [81]. Postmortem examination of patients with CS revealed small blood vessels of pulmonary tissue being occluded with platelets and small thrombi which consequently resulted in diffuse pulmonary damage [68]. Owing to the higher incidence of pulmonary thromboembolism in patients with CS, the hypothesis of hypercoagulability in CS merits further investigation. Recently large cohort study (n = 449) was conducted in CS patients and they found a significant correlation between higher levels of D-dimer and prothrombin time with mortality rate [82]. So, they proposed D-dimer and prothrombin time can serve as early biomarkers of the cytokine storm progression. However, they refuted the use of platelet count and aPTT as markers of the cytokine storm as they could not observe any significant difference between CS patients and the healthy class of patients [83]. These propositions are further supported by recently conducted meta-analysis considering 9 studies and they reported unanimously elevated levels of D-dimer in critically ill patients compared to patients group moderately ill [84]. Other cellular determinant of cytokine storm recognized include significant neutrophilia which has been reported to cause cellular damage to the tissue architecture and causes necrosis of the tissues. Earlier studies conducted in Wuhan have reported significant elevation in neutrophil count in non-survivors compared to patients that survived, furthermore, a steady increase in mature and immature neutrophil levels was observed till the death of these patients [11]. Another study utilized a multi-omics approach and found that markers of neutrophil (Neutrophil Elastase and Myeloperoxidase) were significantly elevated in critically ill patients with cytokine storm compared to healthy counterparts [85]. Another study conducted by Ref. [86] found increased levels of pyruvate kinase M2 (PKM2) an indicator of hypoxia in critically ill patients with cytokine storm compared to patients with mild degree of illness. Furthermore, an immune-metabolomics study found that cytokine storms cause programming and proliferation of neutrophils [33]. These findings are further supported by the characteristic clinical presentation of cytokine storm which includes elevation in an array of cytokines including interleukins (IL) (IL-1, IL-2, IL-6, IL-7, IL-8, IL-10, IL-12, IL-17, and IL-18) [87]. In addition to this, lymphopenia has been observed in CS, indicating that cytokine storm is triggered by innate rather than adaptive immune cells [87].

### 7.1 Clinical presentation as criteria for CS diagnosis

The duration of the CS can be predicted by the etiology of the disorders and their therapeutic interventions used to treat the disease [1]. Almost all patients with CS and respiratory illnesses experience high fever, tiredness, loss of appetite, headache, rash, diarrhea, joint pain, muscle pain, and mental symptoms [70] (Fig. 1). These symptoms could be caused by acute physiological changes, cytokine-induced tissue damage, or immunological reactions mediated by immune cells [88]. They can cause disseminated intravascular coagulation, hypoxemia, hypotension, dyspnea, hemostasis imbalance, and vasodilatory shock henceforth may necessitate invasive ventilation in respiratory diseases [89].

Although detailed guidelines for understanding the above-mentioned changes are yet to be established, henceforth designing a scoring system like that of the Penn grading scale, MS scoring and HS scoring which are established scoring systems developed for the characterization of adverse events may provide some benefit in prediction of COVID-19 CS [20]. In this direction [20], proposed criteria for the characterization of COVID-19 CS based on three cluster models which include (i). Albumin level less than 2.87 mg/mL, lymphocyte count less than 10.2 %, and neutrophil count more than 11.4 × 10^3^/mL. (ii). ALT level more than 60 IU/L, AST level more than 87 IU/L, D-Dimer levels more than 4930 ng/mL, LDH levels more than 416 U/Land troponin I level more than1.09 ng/mL (iii). Anion gap less than 6.8 mmol/L, levels of sodium, potassium, and urea above the reference range values, and ferritin levels more than 250 ng/mL. On similar lines [90,91] proposed revised criteria for diagnosis of CS, which included a fraction of oxygen saturation to a fraction of inspired oxygen levels (SPO_2_/FiO_2_), significantly higher levels of ferritin, C-reactive protein, and D-Dimer. Recently [13] proposed the use of HS scoring in combination with biochemical assay for diagnosis of COVID-19 CS. Despite the need for validation and precision, these studies provide baseline criteria for designing officially accepted guidelines for the fabrication of diagnostic criteria for COVID-19 CS.

## 8 Cell types and signaling pathways involved in pulmonary cytokine storm

**Fig. 2:**
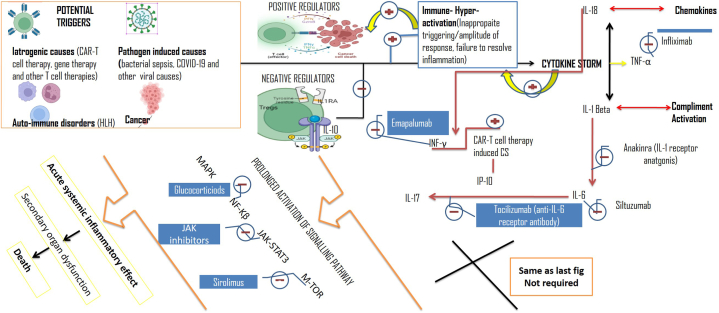
Various sub-cellular pathways involved in CS, these pathways are involved in the promotion of hyper-transcription of inflammatory, pro-inflammatory, and other mediators that fuel the CS.

**Fig. 3:**
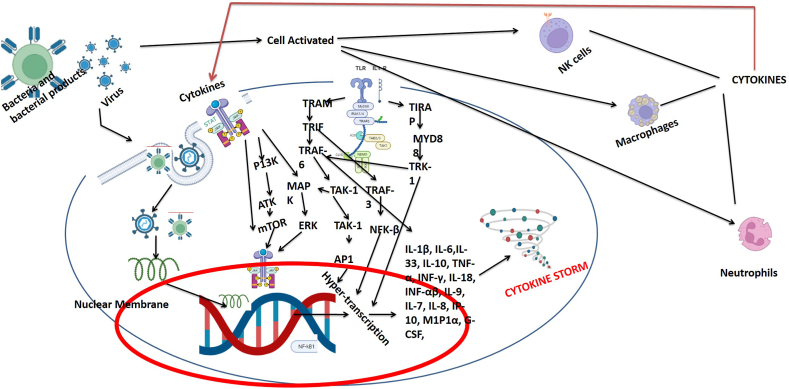
Potential triggers of CS, positive and negative regulators involved in the pathogenesis of CS, and description of effective blockers to ameliorate the progression of CS.

Inflammatory cell types such as lymphocytes, reticuloendothelial cells, and NK cells are all involved in the pathophysiology of CS in respiratory diseases. Neutrophils help to generate thrombi, whereas macrophages derived from circulating monocytes have several functions, including ingestion of foreign materials and initiating an immune response. In a cytokine storm, different immune cells secrete different cytokines, for instance, T cells and NK cells, release IFN-γ, which stimulates macrophages and causes clinical symptoms characteristic of CS [92]. The cytokines released from activated immune cells trigger a cascade of immune signaling pathways causing CS during respiratory diseases. Excessive cytokine release is induced by macrophages, which can result in tissue damage and organ failure in some cases. In patients with lung infections, CS has been found to have hemophagocytic macrophages in their bone marrow. Interferon-γ (IFN-γ) causes macrophage-induced hemophagocytosis, which may contribute to the cytopenia found in CS patients [[57], [58], [59]]. NK cells cause cytolysis, which is decreased in some types of cytokine storm, resulting in prolonged stimulation of antigenic determinants and ineffective resolution of inflammation [60]. In addition to this by reducing perforin and granzyme synthesis by IL-6, NK-cell function is reduced. Hence excessive Th1-type inflammatory responses result in considerable levels of interferon-delayed hypersensitivity reactions and macrophage activation, both of which are required for intracellular infection defense. COVID-19 CS is a dynamic process that involves hyper activation of inflammatory processes and the activation of various signaling pathways. In the subsequent section, we will discuss the related pathways involved in COVID-19 CS (Fig. 2, Fig. 3).

### 8.1 IL-6/JAK/STAT signaling pathway

The signaling pathway was proposed based on the predominant finding of elevated IL-6 levels observed in critically ill COVID-19 patients [61]. Increased levels of IL-6 cause activation of trans and cis signaling of JAK/STAT, which is supposed to have a pleiotropic effect on immune cell activation, reduced synthesis of Tregs, and enhanced recruitment of immune cells and differentiation of CD4^+^ and CD8^+^ T cells [62]. This results in the vicious circle of further production of pro-inflammatory cytokines and acute phase proteins which results in hyper-inflammatory response and organ failure [63].

### 8.2 IFN-γ/JAK/STAT signaling

IFN- γ is the main activator and driver of immune cell proliferation and exerts protection against viral and bacterial infections through up-regulation of JAK1/JAK2 and down-regulation of STAT1-IFN-γ-activated site (GAS) cascades [55]. There is a plethora of studies that postulate hyper-activation of immune and inflammatory pathways but it is not clearly defined how IFN- γ is involved in CS. However, based on its role in immune activation it can be well postulated that IFN- γ has a significant role in CS. Of special interest, some studies have found significantly decreased levels of IFN- γ produced byCD4+ T cells in patients with severe manifestation of disease compared to patients with mild manifestation of disease; these findings have been attributed to functional exhaustion of CD4^+^ T cells [64]. So, it has been postulated that elevated levels of IFN- γ occurs from macrophages and not from CD4^+^ T cells [65].

### 8.3 TNFα/NF-κB signaling

TNFα is one of the most important pro-inflammatory cytokines produced by immune cells on their activation [66]. For the production of TNFα and other pro-inflammatory cytokines, NF-κB plays a pivotal role via activation of pro-inflammatory and anti-apoptotic genes [67]. Henceforth, TNFα/NF-κB interplay causes initiation of CS which subsequently causes apoptosis of the epithelial cells and results in epithelial-immune cell interplay to fuel inflammatory processes [93]. Contrary to this hypothesis, a clinical trial conducted in Wuhan China found significantly reduced levels of TNFα in critically ill patients compared to moderately ill patients [94] and subsequent studies reported levels of TNFα within the normal range in different patients’ classes across the severity of disease [95]. Hence there is an urgent need for further research to understand the role of TNFα in CS, although recent research suggests that inhibition of the TNFα/NF-κB signaling pathway causes improvement in critically ill COVID-19 patients [96].

### 8.4 NLRP3/IL-1β signaling

An important pro-inflammatory cytokine secreted by macrophages causes migration of immune cells to the infected site, production of various adhesive factors, and has positive feedback on activation of NF-κB and results in auto-activation for production of more IL-1β quantity [97]. It has been proposed that excessive ROS (Reactive Oxygen Species) production causes activation of NLRP3 which results in cleavage of inactive IL-1β precursor into an active form of IL-1β, hence initiating a vicious circle of cytokine storm [98]. Owing to the increased incidence of cytokine storm in COVID-19 patients there have been significant improvements and enhancements in research on cytokine storm-associated COVID-19 and various signaling pathways have been proposed. These pathways are indicated in Table 3.

## 9 Cytokines involved in pulmonary CS

Among the cytokines involved in the CS, the most prominent are recombinant cytokines and interferon [[29], [30], [99]]. Key cytokines of CS include INF-γ, IL-1, IL-6, TNF, and IL-18, which are assumed to serve crucial immunopathology activities [100]. IL-1, IL-6, and TNF-α produce fever, which is a clinical characteristic of cytokine storm. IL-6, a critical modulator of the acute inflammatory response and a CS pathophysiological characteristic is greatly increased in a variety of immunopathology illnesses [[13], [101], [102]]. Among the various pro-inflammatory cytokines, the following pro-inflammatory cytokines deserve special mention (Table 3).

### 9.1 IL-6

IL-6 is one of the pivotal cytokines because it is produced by numerous organ systems and has effects on both immune and non-immune cells. The *cis*- and *trans*-signaling routes are the two main signaling pathways [103] involved in the production of IL-6. Membrane-bound gp130 is found throughout the body, but membrane-bound IL-6 Receptors are found mostly in reticuloendothelial cells. The pleiotropic consequences of cis signaling activation on the immune system cause CS [103]. TNF-α is a multifunctional inflammatory cytokine that produces fever, increases systemic inflammation, activates antimicrobial responses like IL-6, and regulates immunity and cell death. It is classified under the TNF–TNF receptor superfamily. TNF activates NF*k*-B, which stimulates many genes involved in inflammatory response.

### 9.2 IL-18

Furthermore, in addition to these mediators, IL-18 has also been linked to CS [71]. The alternate pathway involves pathogenic microorganisms and sterile stressors, which are detected by inflammasomes, which are multi-molecular cytosolic sensors. Caspase-1 is activated during pyroptosis, which then activates IL-18 and IL-1 from their progenitors [104,105]. Recently some of the studies have found that IL-18 activatesTh1-type responses by stimulating the release of INF-γ from immune cells. It produces Th1-type inflammatory responses in synergism with IL-12 and IL-15. In individuals with CS-induced immune cell stimulation syndrome, blood concentrations of IL-18 are high [[106], [107], [108]].IL–18–binding protein (IL18BP) controls the pro-inflammatory effects of IL-18 and aids in the prevention of IL-18 and receptor binding [104,109].

### 9.3 IL-10

To decrease systemic off-target effects, regulatory cytokines like IL-10 and naturally occurring pro-inflammatory cytokine antagonists like IL1RA are used as immune modulators. TNF, IL-1, IL-6, and IL-12 are all inhibited by IL-10, as well as antigen presentation is also inhibited by IL-10 [110].

### 9.4 Complement proteins

Plasma proteins like complement proteins and some less understood proteins act as promoters to cause CS by providing feedback on cytokine signaling, detecting infections, and enhancing cellular responses. The production of complement proteins is increased by cytokines, which maintains the feedback mechanism by increasing or lowering cytokine production. Complement, although its high efficacy at killing microorganisms, can occasionally cause collateral damage if created in excess. To support these propositions Hypocomplementemia has also been discovered in situations of CS caused by the overconsumption of immune complexes [111]. A significantly positive correlation was found between levels of pro-inflammatory cytokines (IL-1β, IL-6, IL-8, and TNF-α) and complement factors (C3a, C5a, and factor P (properdin) in critically ill COVID-19 patients with associated CS compared to moderately ill patients, henceforth these findings reflect the potential utility of complement factors to serve as biomarkers of severity of disease progression in COVID-19 associated CS [13].

## 10 Types of pulmonary cytokine storm

### 10.1 Iatrogenic cytokine storm

Infusion of engineered CAR T cells into CD19^+^ lymphoma patients triggers a CS by boosting IFN-γ and IL-6 levels [[111], [112], [113]]. Pre-clinical studies have found that macrophages play a pivotal role in the progression of CS by releasing cytokines and promoting immune dysfunction and these immune dysregulations can be effectively reversed by IL-1 [114]. Similarly, iatrogenic CS has been observed as a result of tumor lysis produced by the introduction of pyroptosis in target tumor cells [115]. Immunotherapy which activates T cells by binding to both CD19^+^ and CD3^+^ T cells results in iatrogenic CS and the pathological cascade has been successfully inhibited by using anti-IL-6 antibody therapy, which further affirms the role of IL-6 [97]. T-cell stimulation triggers iatrogenic cytokine storm, which is propagated by immune cell activation. CD28 receptor is a secondary receptor located on the T cell membrane after the primary receptor of T Cell Receptor (TCR), these two receptors play pivotal roles in the activation of T cells, which include T cell differentiation and T cell remodeling and immune modulation and immune regulation. Studies have found that infusion of ant-CD28 (TGN1412) results in an immune imbalance which triggers hyper-activation of immune cum inflammatory response and culminates into CS [116]. This is because of the simultaneous stimulation of diverse sets of immune cells [117]. Although CAR T cells or Blinatumomab contribute to the cytokine storm, this is not necessarily the case in all patients due to other factors which include genetic background of patients, dose of the drug, and time of introduction/initiation of therapy [118,119]. Other iatrogenic causes of CS include drugs obtained from biological organisms like Rituximab [96], gene therapies, immune system hotspot inhibitors, organ transplant surgeries [120], and allogeneic stem-cell transplantation, in addition to these bioterrorism agents like Staphylococcal enterotoxin B and *Francisella tularensis* also cause iatrogenic CS [96].

### 10.2 Pathogen-induced pulmonary cytokine storm

Despite the paucity of evidence, investigations have demonstrated that pathogen-induced pulmonary CS can be caused by a diverse set of pathogenic micro-organisms [121,122]. Sepsis in microbial infections can result in fever, cell death, coagulopathies, and multi-organ failure due to cytokine production [123,124] (Table 1). Excess cytokine production can cause more harm than infection, therefore it can have a more detrimental impact [125].

#### 10.2.1 Bacterial infections

Super-antigens produced by bacteria such as *Streptococcus* species and *Staphylococcus aureus* bind with the MHC and T-cell receptors, resulting in T-cell stimulation and concurrent release of cytokines which culminate into toxic shock syndrome [126]. These super antigens are strong T-cell mitogens, capable of causing fever, shock, and death at doses as low as 0.1 pg/ml [119].

#### 10.2.2 Viral infections

It has been found that widespread viral infections can also set off cytokine storms. A high inflammatory response against infectious agents has been shown to alter pathogen detection, effectors, regulatory systems, and hyper-activation of inflammation [57,127]. Furthermore, studies have reported multi-centric Castleman's disease (HHV-8) caused by Kaposi's sarcoma herpesvirus as an important disease that involves CS in its pathogenesis [128]. From cell line studies it has been found that HHV-8 infected cells produce significantly higher levels of interleukin-6 which results in a CS [129,130]. By similar pathways viruses such as herpes simplex virus and influenza viruses such as H5N1 can also induce CS [131].

### 10.3 Monogenic or autoimmune cytokine storm

This type of CS is caused due to impairments in recessive autosomal genes. For instance, in patients with primary HLH granule-mediated cytotoxicity has been attributed to recessive mutation in autosomal genes [132]. In addition to this, patients with secondary HLH, experience CS as a result of a variety of pathogenic, immune-mediated, or metaplastic disorders, and mutations in the selected genes [133,134]. In HLH, the levels of cytokines are elevated [132]. In some HLH patients, the beneficial effects of immune-suppressants, anti-neoplastic drugs, anti-inflammatory antibodies, pro-inflammatory antagonists, IL-6 antagonists, and anti-inflammatory drugs point that the pathogenic hotspots targeted by these medications have a significant role in the progression of auto-immune pulmonary CS [16]. Humans affected with this disorder have structural changes in genes influencing the immune system and inflammasome stimulation [135]. Such patients experience unprovoked release of cytokines and initiation of CS without signs of infection. Based on the studies that have postulated genetic defects as possible risk factors for the pathogenesis of this disorder, these studies have reported TNF-α, IL-1, and IL-18 as major drivers of cytokine storms in these genetic diseases [127]. Chronic granulomatous disease and STAT1 gain-of-function disease are two hereditary immunodeficiency illnesses that have been associated with CS [136]. The CS was most severe and was manifested as thrombocytopenia, anasarca, fever, reticulin fibrosis, and organomegaly (TAFRO) subtype [137]. In the wide spectrum of auto-immune pulmonary CS instances, IL-6 was found to be the primary driver of pathogenesis, while the rationale for this is unknown [138]. So recent studies have postulated that CS in respiratory illnesses promotes pathologic changes via engaging macrophages and/or monocytes [20]. These changes lead to hyper inflammatory response in respiratory diseases, with the formation of free radicals which subsequently cause cell death [139]. Henceforth the ROS (Reactive Oxygen Species) promotes the synthesis of NLRP3 and NF-kB, resulting in an increase in cytokine production and, eventually, a CS [61]. Ultimately these patients develop ARDS, sepsis, multiple organ dysfunctions, and as a result, eventually die of auto-immune pulmonary CS [140].

## 11 Relationship between inflammatory cytokine levels and pulmonary disease progression

A Plethora of preclinical and preclinical studies have investigated the casual relationship between elevated inflammatory cytokine levels and the progression of pulmonary diseases, with special emphasis on chronic respiratory conditions such as chronic obstructive pulmonary disease (COPD) [31], asthma [141], acute respiratory distress syndrome (ARDS) and interstitial lung diseases [142]. In these respiratory diseases, the role of dysregulated cytokine responses in driving lung pathology has been identified as a prominent pathology [143]. Recent investigations into the COVID-19 pandemic have further emphasized the critical interplay between inflammatory cytokines and pulmonary disease progression [144]. Understanding the dynamics of inflammatory cytokines in pulmonary diseases is crucial for the development of targeted therapeutic interventions. Interventions aimed at modulating cytokine levels, such as the use of anti-cytokine antibodies or small molecule inhibitors, are areas of active research and may hold promise for mitigating pulmonary disease progression [145].

So, researchers have investigated the molecular determinant activated by pathogens and they have identified various cellular and subcellular moieties. Pathogens encounter a robust immune response orchestrated by the host's defense mechanisms, a critical element in combating infections [146]. Pattern recognition receptors (PRRs) on host cells play a pivotal role in identifying a broad spectrum of cellular surface moieties on pathogens, termed "pathogen-associated molecular patterns" (PAMPs). The interaction between PAMPs and PRRs initiates an inflammatory cascade, triggering the activation of various products, including genes encoding pro-inflammatory cytokines [92]. Key mediators, such as NF-kB, activation protein 1, and interferon response factors 3 and 7, activated by PRRs, drive the expression of potent inflammatory mediators [73]. Consequently, immune cells and proteins migrate to the infection site, resisting the invading pathogens. Elevated cytokine levels facilitate immune cell recruitment from the bloodstream, leading to the destabilization of endothelial cell-cell connections, capillary endothelial damage, and eventual diffuse alveolar damage [147]. Cytokine storm (CS) inflicts severe damage to pulmonary tissue, potentially progressing to Acute Respiratory Distress Syndrome (ARDS), representing the most devastating consequence of CS [[17], [148], [149]]. Immediate CS treatment is imperative to prevent ARDS, as a lack of adequate medication may result in multiple organ dysfunctions and fatalities. Recent findings, notably in the context of the COVID-19 pandemic, underscore the respiratory infection's severe impact on various organs, particularly the lungs. Post-mortem examinations of lung tissue in severely affected COVID-19 patients reveal extensive alveolar edema, fibroblast infiltration, fibrin deposition, and lymphocytic infiltration [55]. Critically ill COVID-19 patients exhibit significantly higher concentrations of interleukins, TNF-α, and immune attractant proteins (protein 1 and protein 1A) compared to mildly infected cases [[18], [22], [23]]. This underscores the importance of understanding and addressing cytokine responses, highlighting potential therapeutic interventions to mitigate the severity of respiratory infections and improve patient outcomes.

## 12 Clinical course of cytokine storm

The clinical course of a cytokine storm typically involves distinct phases, ranging from initiation to resolution or, in severe cases, progression to organ failure [43].

i. **Initial phase:** This phase is also called as prodromal phase, which is characterized by the activation of immune cells in response to an event like a viral infection, microbial infection, or other inflammatory stimuli [24]. As a result of the production of multiple pro-inflammatory molecules, lung epithelial cells, pulmonary parenchyma, and associated pulmonary micro-vascular architecture undergo apoptosis, resulting in alveolar edema [25]. Chronic manifestation of CS results in pulmonary fibrosis mediated by pro-inflammatory factors as well as chemokines and reactive oxygen species [26].
ii. **Amplification phase:** This phase is characterized by excessive release of pro-inflammatory cytokines (IL-6, TNF-α IL-1β) which contributes to the systemic nature of the CS and can lead to widespread inflammation and tissue damage [27]. The CS can disrupt endothelial cell function, resulting in coagulation abnormalities, micro vascular permeability alterations, hemorrhages, and thrombosis [28]. Furthermore, extravasations of solutes, fluid, macromolecules, and hormones, as well as platelets and blood cells are facilitated by endothelial cells. In normal circumstances, epithelial cells use a surface enzyme ecto-ATPase to break down adenosine triphosphate (ATP) and adenosine diphosphate (ADP) in healthy people. In CS and likewise conditions epithelial cell mal-function leads to abnormal ADP production and platelet activation, which leads to thrombosis. This initiates a vicious cycle whereby abnormally high concentration of ADP causes inactivation of surface enzyme ecto-ATPase henceforth further inhibits hydrolysis of ATP and ADP molecules [150].
iii. **Organ Dysfunction Phase:** This phase of CS leads to organ dysfunction and this phase is often associated with life-threatening complications and requires intensive medical intervention [151]. Overall, CS manifests clinically as "overlap syndrome" marked by a significantly reduced cell count, reduction in ESR, elevated ferritin level, NK dysfunction, and enhanced auto-hematophagy [152], which leads to increased perivascular cupping of activated immune cells. Furthermore, this causes recruitment of fibroblasts in pulmonary tissue which results in widespread fibrin deposition which leads to alveolar collapse [153,154].
iv. **Resolution Phase:** In some cases, CS may resolve spontaneously as anti-inflammatory and immune modulatory mechanism comes into play. However, this phase is highly dependent on the specific context and the underlying cause of the cytokine storm

In the following section, we will be discussing the involvement of the CS in some of the prominent viral respiratory pandemics in detail. Furthermore, their presentation is tabulated in Table 1, Table 2.

### 12.1 COVID-19

In recent studies conducted throughout the globe, circulating pro-inflammatory cytokine levels were found to be positively correlated with the severity of diseases in COVID-19 [155]. These pro-inflammatory cytokines have been linked to the cause of mortality in COVID-19-infected individuals [156]. For instance, CS in COVID-19 can be attributed to the stimulation of immune receptors that are membrane-bound and which drive Th1 cells, CD14^+^ and CD16^+^ monocytes [157]. The pathogenic Th1 lineage is swiftly stimulated by SARS-CoV-2, causing the release of IL-6 and granulocyte-macrophage colony-stimulating factor (GM-CSF) [158]. GM-CSF also causes inflammatory CD14^+^ and CD16^+^ monocytes to become activated and production of IL-6 and TNF-α. Toll-like receptors (TLRs) and F care types of membrane-bound immune receptors also contribute to dysregulated inflammatory response, furthermore, insufficient IFN-induction can lead to increased cytokine output [159]. Recently an important finding has been reported which postulates that in COVID-19, cytokine release is triggered by extracellular traps of neutrophils [160]. CSIL-6 performs an important function by increasing vascular permeability which henceforth results in interstitial edema induced by activation of the complement system (CS) and allowing mast cells to release histamine [161]. Furthermore, the coagulation pathway is activated by IL-6 which can lead to DIC (Disseminated Intravascular Coagulation) [162]. In patients infected with COVID-19, recent research has attributed the grave prognosis of disease to cytokine storms as well as other factors such as old age, corticosteroid medication, and immune-competence [64].

### 12.2 MERS (Middle east respiratory syndrome)

MERS infection is associated with significantly enhanced levels of pro-inflammatory cytokines [[162], [163], [164]]. Similarly, it was found that antiviral cytokines tend to decrease in MERS while pro-inflammatory cytokines, particularly IL-6, increase in these individuals [165]. The clinical manifestations of the disease which include deadly acute respiratory failure, pulmonary fibrosis, and collapsing alveoli, are attributed to CS [166]. Several mediators appear to be the most active mediators in the onset of extreme respiratory failures, as these factors diffuse into tissue substratum and cause tissue damage [19]. Furthermore, some chemokines and cytokines as well as induced protein 10 (IP10), MCP-1, IL-6, and IL-1 were considerably enhanced in severe COVID-19, MERS, and SARS, according to recent investigations [[167], [168], [169]].

### 12.3 H1N1 Influenza A

These viruses proliferate in pulmonary epithelial cells which create a “cytokine storm" of inflammatory cytokines/chemokines, which is eventually activated by viral nonstructural protein 1 (NS1) [170]. The pathophysiology and poor clinical outcomes are mostly caused by uncontrolled replication of the virus which subsequently causes CS [65,171]. When the H_2_O_2_-MPO system is activated, the NS1 type of viral protein produced by the cells promotes excessive production of MCP-1 by macrophages and IL-8 a chemokine by neutrophils implying that the NS1viral protein of influenza virus H1N1 (PR-8) plays a key role in the CS [59]. The CS induced by the influenza virus NS1 is considered a primary regulator and it has been verified in animal models [172]. The CS does not appear to be caused solely by the infiltration of macrophages and NK cells. The innate immune cells are driven to the lung which intensifies the cytokine storm, increasing lung damage. The elevated concentrations of cytokines, serum interferon, and other mediators of inflammation in individuals with H1N1-induced pneumonia and ARDS are indicative of a CS [173].

## 13 Therapeutic options available

**Fig. 4:**
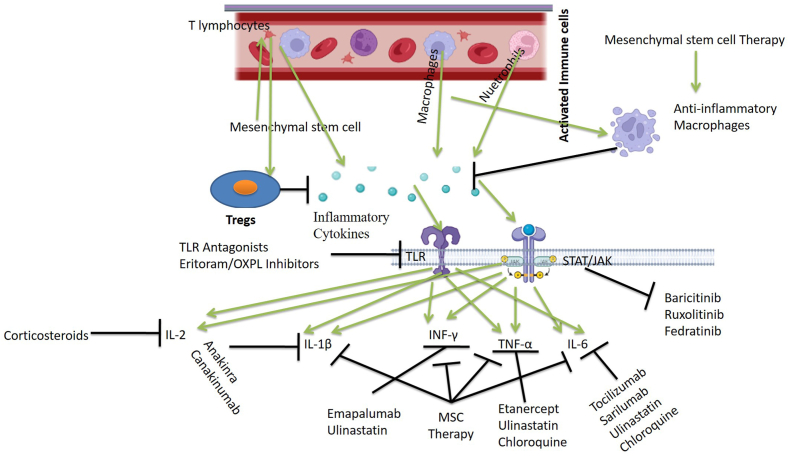
Therapeutic options available for the treatment of cytokine storm in respiratory disease with a special focus on targeting potential hotspots involved in the progression of CS.

CS results in high death and grave prognosis henceforth early treatment of viral infections like MERS and SARS [174], as well as the use of immune-modulators to control inflammatory responses [175] has been shown to enhance the prognosis [[176], [177], [178], [179], [180]] (Fig. 4; Fig. 2; Table 4).

**Table 4:** Therapeutic interventions for the treatment of cytokine storm in respiratory disease.

Interventions	Role	Benefits	Drawbacks	References
Hydroxychloroquine/Chloroquine (n = 166)	Inhibits the production of IL-6 and TNF	Reduce viral load and infection duration	Retinopathy, myopathy, neuromyopathy, cardiopathy, and arrhythmia	[64]
IL-6 Receptor Inhibitors (tocilizumab) (n = 21) (n = 3924)	Blocks IL-6 receptors and hence prevents interaction of IL-6 with its cellular receptors	Improved clinical outcome Significantly reduced need for invasive ventilation Decreased hospitalization Significantly reduced mortality	Not well known	[207]
IL-1 β Blocker anakinra (N = 22)	These drugs bind with IL-1 and causes competitive inhibition of the IL-1β	Decreased serum levels of Ferritin, C reactive protein Improved clinical score of patients	Not well known	[208]
IFN-λ blocker Emapalumab (trail has been terminated)	These activates anti-viral genes in epithelial cells	Causes stimulation immune system without causing over activation of immune response	Treatment was not able to reduce mortality and at later stages of disease drug was in-effective	209
IL-6 inhibitors (siltuximab) (n = 30)	Inhibits IL-6 and hence its interaction with IL-6 receptor	Improvement in clinical outcome of more than 50 % of patients	Not well known	[210]
IL-1 Family Antagonists	Caused immune-modulation	Significantly reduced mortality during hospital stay of 28 days	Needs to be evaluated through clinical trials and animal studies	[211]
Corticosteroids Retrospective study (n = 401)	Suppress the host inflammatory and immune reaction	Reduces mortality and stay in hospital by 3–5 days Lower incidence of secondary infections	impaired viral clearance in non-ICU patients avascular necrosis, diabetes, and psychosis	[212]
TNF-blockers infliximab (n = 56)	Ameliorates T cell response and causes restoration of lung injury	Significantly improved survival of patients with systemic sepsis.	Limited number of patients have been observed but study need to be observed in large clinical trial	[7]
Sepsivac (Mycobacterium Indicus Parnii) (n = )	Acts as immune modular and keeps effective check on activation of inflammatory pathways	Reduced days of hospitalization and need for mechanical ventilation	No side effects were observed except increase in levels of ALT and AST.	[213, 214]
Tocilizumab (n = 122)	Blocks IL-6 signaling pathway to prevent cytokine storm. Was found to be effective in patients with bilateral lung lesions	Improves survival outcome Decreased days under mechanical ventilation	Severe infections, neutropenia, hepatic damage, and thrombocytopenia	[209]
GM-CSF inhibition Sargramostim/Molgramostim (n = 13)	Cause modulation of immune system	Rapid clinical improvement, restoration of blood biochemistry to normal and pyrexia resolution	Not well known	[210]
MSC	Prevents the aberrant stimulation of macrophages and T cells and the release of proinflammatory cytokines	Decreases mortality	Not well known	[42]
Anakinra	Inhibits IL-1α and IL-1β	Improves respiratory function and increases patient survival rate	Increases bacterial infection risk	[100]
Intravenous immunoglobulins (n = 99)	Block Fc receptors and exert different immunomodulatory effects	Reduces severity of inflammation immune substitution and immunomodulation	Thrombosis, Lung injury	[211]
Ulinastatin	Decreases IL-6, IFNα and TNF while increases IL-10 levels	Shield the vascular endothelium by preventing inflammatory mediators from being produced and released	Femoral head necrosis	[207]
JAK/STAT inhibitors (N = 56)	Inhibit inflammatory cytokines and decrease viral entry into cells	Improve clinical symptoms and respiratory parameters	Inhibiting the production of IFNα which is important for fighting viruses	[206]
NF-κB pathway blockers	Preclinical studies have reported that blocking NF-κB causes immune modulation	Preclinical studies support its use in randomized clinical trail	Not well known	212

### 13.1 IFN-λ

IFN- λ has been employed to cure CS by activating epithelial cells, which reduces IFN-'s pro-inflammatory effect mediated by mononuclear macrophages [170]. Additionally, it aids in preventing neutrophils from being assigned to inflammatory areas [171]. The antiviral genes are activated by IFN-λ in epithelial cells, resulting in antiviral actions without over stimulation of the immune system [70]. Interferon lowers viral load efficiently when given early and hence improves patients' clinical symptoms to some degree. Interferon, however, has failed to control death rates [62] (Fig. 4; Fig. 2).

### 13.2 Corticosteroid therapies

Corticosteroids have anti-inflammatory properties and are widely used to reduce inflammation [181]. Early benefits such as a decrease in fever, relief from lymphocytic infiltration in pulmonary tissue [172], and improvement in oxygenation have been noted if corticosteroids are administered appropriately and on time [172]. Corticosteroid-treated SARS patients had lower death rates, and subsequent infections and associated problems were uncommon in them [173]. However, investigations have demonstrated that giving corticosteroids to human SARS-CoV patients has negative consequences. In lung infections like COVID-19, corticosteroid therapy is still a challenge for clinicians [174], because timely administration of corticosteroids and adequate dosage is critical. If glucocorticoids are given too early, the body's immunological defense mechanism is hindered, which leads to an increase in viral load and ultimately unfavorable effects. As a result, glucocorticoids are used primarily in the treatment of critically ill patients who are experiencing an inflammatory cytokine storm. Excessive inflammation is controlled in the early stages of an inflammatory CS when glucocorticoids are given on time, which successfully prevents ARDS and protects organ functions in patients. In patients with significantly reduced oxygen saturation, deteriorated imaging, and an exaggerated inflammatory reaction, glucocorticoid therapy within 3–5 days has been proven to be beneficial. However, because of the immunosuppression caused by high dosages of glucocorticoids, the clearance of coronavirus can be slowed [[175], [176], [177]] (Fig. 2).

### 13.3 IL family antagonists

#### 13.3.1 IL-1 antagonists

The important pro-inflammatory markers involved in CS are IL-1, IL-6, and IL-33 [178]. Studies that attempted to ameliorate CS by inhibiting IL-1 have received a lot of attention. An IL-1 blocker, Anakinra, resulted in an improvement in the 28-day survival rate of seriously ill COVID-19 individuals with extreme sepsis when treated for infection-induced CS [58]. There is inadequate data that supports the use of specific IL-1 class antagonists for the treatment of COVID-19, which requires additional verification in actual clinical settings using randomized case-control clinical studies [179] (Fig. 2, Fig. 4).

#### 13.3.2 IL-6 antagonists

Tocilizumab, being an effective IL-6 antagonist has been used against autoimmune illnesses as it helps in suppressing the immune system [180]. Furthermore, Tocilizumab is a drug that is used to treat infection-induced cytokine storms [182]. In the case of pulmonary infections like COVID-19, serum IL-6 levels were found to rise dramatically in seriously ill individuals. Clinical trials of Tocilizumab in China recorded that it is highly efficient in curing critically ill subjects with severe pulmonary lesions and increased IL-6 levels [183,184].

### 13.4 Pro-inflammatory cytokine blockers

#### 13.4.1 TNF-α blockers

TNFs are major inflammatory mediators and interesting targets for CS management. The survival rates were considerably improved in patients with sepsis who received anti-TNF medication as per [90] as well as effective in the treatment of non-infectious disorders such as atherosclerosis [185].In animal models, TNFs have been associated with acute lung injury, and TNFs have also been shown to block T cell response in humanized laboratory animal models of SARS-CoV [186]. Mice were found to be protected from SARS-CoV-related morbidity and mortality when TNF activity was neutralized or the TNF receptor was deleted [187] (Fig. 2, Fig. 4).

#### 13.4.2 IFN-αβ inhibitors

IFN inhibits virus multiplication by activating the IFN-αβ stimulated gene. IFN-αβ, on the other hand, has been found to exacerbate illnesses by increasing the stimulation of cells of innate immunity and mononuclear macrophages [188]. Early interferon signaling has been proven to be protective in humanized laboratory animal models of respiratory infection, whereas chronic IFN signaling has been shown to cause an imbalance of anti-SARS-CoV immune responses in humans [47]. To minimize excessive inflammatory reactions, these antagonists should always be used in the early stages of serious illness [52].

### 13.5 Chloroquine

Chloroquine is an anti-malarial medication that was discovered in 1934 and is included in the WHO's Model List of Essential Medicines for 2019 [71]. Chloroquine inhibits the production of pro-inflammatory cytokines, implying these mediators can ameliorate the CS in clinical cases of pulmonary infections. Chloroquine phosphate has also been utilized in China to treat adults aged 18 to 65 infected with COVID-19 [98].

### 13.6 Ulinastatin

Ulinastatin belongs to the category of natural anti-inflammatory drugs that shield the vascular endothelium by preventing inflammatory mediators from being produced and released [189]. It is used against pancreatitis and acute circulatory failure [56]. Ulinastatin helps to reduce those cytokines that fuel the CS process (TNF, IL-6, and IFN-α) and simultaneously enhances levels of that cytokine which dampens the inflammatory process (IL-10). In humans, Ulinastatin maintains the equilibrium between these two classes of cytokine responses by preventing CS hence regulating inflammation's chain reaction [21]. Several animal studies have shown that high-dose Ulinastatin has an anti-inflammatory effect similar to glucocorticoid hormones. Ulinastatin, differs from glucocorticoids, as this drug does not suppress immune response processes and is unlikely to induce side effects most commonly found in glucocorticoid therapy, for instance, femoral head necrosis. These drugs are frequently employed in the treatment regimen for COVID-19 individuals.

### 13.7 Oxidized phospholipids (OxPL) inhibitors

Oxidized phospholipids inhibitors were reported to ameliorate CS in humanized animal models of respiratory infections by decreasing the release of cytokines/macrophages in pulmonary tissue via the Toll-like receptor 4 (TLR4) and simultaneously causing induction of INF-γ signaling pathway [53]. TLR4 antagonist Eritoran has high immune modulatory properties but no direct antiviral action. Eritoran decreases the synthesis of OxPL, inflammatory mediators in humanized animal models, resulting in a reduction in mortality [34]. Human coronaviruses have been reported to generate enhanced OxPL production in the lung tissues of patients, leading to ALI (Acute Lung Injury) [53]. The Eritoran and other OxPL inhibitors, as a result, appear to be effective in reducing HCoV-induced inflammation.

### 13.8 Sphingosine-1-phosphate receptor 1 (S1P1) agonist therapy

These drugs belong to the class of signal lysophospholipid that boosts the production and secretion of cytokines [34]. The CS induced by influenza infection leading to tissue damage as a consequence of adaptive as well as innate immune responses [59] is dramatically reduced by the S1P receptor signaling pathways [35]. In a variety of laboratory animal models of respiratory infections, S1P1 signaling in pulmonary tissue has been demonstrated to have beneficial effects against inflammatory responses [59]. As a result, S1P1 agonists act on pathogenic hotspots in the pathogenesis of pulmonary infections and reduce mortality by preventing uncontrolled recruitment of mediators of inflammation and chemokines [59,35]. As SARS-CoV-2 primarily causes disease in epithelial and endothelial cells of the human lung, antagonists of S1P1 may be viable therapeutic medications to reduce inflammatory mediators in HCoV patients with triggered hyper-immune response of cells [59]. Recently these classes of drugs were approved in 2019 to treat multiple sclerosis. However, more research is needed under clinically controlled randomized design to determine whether siponimod is a good substitute for CS therapy in actual clinical settings.

### 13.9 Stem cell therapy

MSCs (Mesenchyme stem cells) are the type of progenitor cells that have powerful anti-inflammatory and immunological regulatory properties in addition to the ability to self-renew and multi-directionally differentiate ability [36]. MSCs promote the development of regulatory T lymphocytes and macrophages, as well as the reduction of aberrant T lymphocyte and macrophage recruitment and stimulation, and regulate the release of mediators of inflammation, henceforth lowering the incidence of hyper-stimulation of the inflammatory pathway [190]. MSC can also produce hepatocyte growth factor (HGF), keratinocyte growth factor (KGF), VEGF, and IL-10, which can ameliorate ARDS, repair lung tissue, and prevent fibrosis [72] (Fig. 4).

### 13.10 Blood purification treatments

Ultra-blood purification procedures can eliminate inflammatory components from circulatory blood to some extent. It involves exchange, adsorption, plasma perfusion, blood/plasma filtration, and other procedures that remove inflammatory components and obstruct the "cytokine storm" [69]. In the initial phase of the disease, blood purification procedures can be used to help severely ill subjects. Academician Li Lanjuan's artificial liver technology can remove inflammatory substances from the blood. Transfusion methodology is being utilized to avert the H7N9 CS, and its deployment in COVID-19 indicates encouraging results [66].

### 13.11 Targeting mononuclear macrophage

The regulation of C–C chemokine receptor type 2 (CCR2) by small interfering RNA (siRNA) has been shown to diminish the transport of inflammatory cells to infection sites, which has also been proven in animal studies [193, 194]. The pulmonary tissue of COVID-19 patients had substantial infiltration of inflammatory cells, according to autopsy studies [110]. When mononuclear macrophages come into contact with single-stranded RNA (ssRNA) viruses, TLR7 agonists induce them to produce a powerful inflammatory response. So using TLR7 antagonists appears to reduce the inflammatory CS caused by SARS-CoV-2 infection. Recently researchers have proposed the strengthening of endothelial barrier as a strategy to avert cytokine storm. Significantly enhanced vascular permeability is an indicator of a modification in the cytokine storm's mechanism. It was discovered in animal studies with sepsis where this strategy activated the endothelial Slit-Robo4 pathway and hence significantly reduced vascular permeability, therefore decreasing infection-induced cytokine storms [69].

## 14 Conclusion

Inflammation plays a pivotal role in robust immunity against any infection after the infectious agent has been identified. When a foreign pathogen enters the body, immune cells are activated, assisting in its eradication and restoring equilibrium. Infected persons with pulmonary involvement experience CS, which results in deleterious effects caused by CS. The use of immunomodulators and cytokine antagonists to control the CS early on, as well as limiting inflammatory cell recruitment in the lungs, is crucial. This increases the efficacy of CS treatment and reduces the number of people who die from the disease. There is an urgent need to identify the mechanistic pathways involved in CS identification of potential hotspots and effective targeting of these hotspots. Although a wide spectrum of medicinal preparations have been proposed to be used against CS, these preparations need to be evaluated in case-controlled randomized clinical trials, so that an effective and precisely personalized therapeutic regimen can be developed against CS in general and pulmonary determinants in particular.

## Data availability statement

The data supporting this study's findings are available from the corresponding author upon reasonable request.

## CRediT authorship contribution statement

**Shahana Riyaz Tramboo:** Writing – original draft, Conceptualization. **Ahmed M.E. Elkhalifa:** Funding acquisition, Conceptualization. **Syed Quibtiya:** Writing – review & editing. **Sofi Imtiyaz Ali:** Writing – original draft, Conceptualization. **Naveed Nazir Shah:** Writing – review & editing. **Syed Taifa:** Writing – review & editing, Data curation. **Rabia Rakhshan:** Writing – review & editing, Formal analysis. **Iqra Hussain Shah:** Writing – review & editing, Investigation. **Muzafar Ahmad Mir:** Writing – review & editing, Methodology. **Masood Malik:** Writing – review & editing, Supervision. **Zahid Ramzan:** Writing – review & editing, Formal analysis. **Nusrat Bashir:** Writing – review & editing, Methodology. **Shubeena Ahad:** Writing – review & editing, Visualization. **Ibraq Khursheed:** Supervision. **Elsharif A. Bazie:** Writing – review & editing, Funding acquisition. **Elsadig Mohamed Ahmed:** Funding acquisition, Formal analysis. **Abozer Y. Elderdery:** Investigation, Funding acquisition. **Fawaz O. Alenazy:** Methodology, Funding acquisition. **Awadh Alanazi:** Investigation, Funding acquisition. **Badr Alzahrani:** Methodology, Funding acquisition. **Muharib Alruwaili:** Validation, Funding acquisition. **Emad Manni:** Methodology, Investigation. **Sanaa E. Hussein:** Writing – review & editing, Funding acquisition. **Ezeldine K. Abdalhabib:** Funding acquisition, Formal analysis. **Showkat Ul Nabi:** Writing – original draft, Conceptualization.

## Declaration of competing interest

The authors declare that they have no known competing financial interests or personal relationships that could have appeared to influence the work reported in this paper.
